# Association between HIV infection and arterial stiffness: A population-based cross-sectional study from Rakai, South Western Uganda

**DOI:** 10.1016/j.athplu.2026.100559

**Published:** 2026-03-20

**Authors:** Charles Batte, Martha Sarah Namusobya, Shivan Nuwasiima, Zangin Zeebari, William Checkly, Godfrey Kigozi, Larry Chang, James Kayima, Petter Ljungman, Helena Nordenstedt

**Affiliations:** a Lung Institute, Department of Medicine, Makerere University College of Health Sciences, Kampala, Uganda; b Department of Global Public Health, Karolinska Institutet, Stockholm, Sweden; c Division of Pulmonary and Critical Care, Johns Hopkins University, Baltimore, MD, USA; d Rakai Health Sciences Program, Entebbe, Uganda; e Makerere University School of Public Health, Kampala, Uganda; f Division of Infectious Diseases, Department of Medicine, Johns Hopkins School of Medicine, Baltimore, MD, USA; g Department of Medicine, Makerere University College of Health Sciences, Kampala, Uganda; h Institute for Environmental Medicine, Karolinska Institutet, Stockholm, Sweden; i Department of Cardiology, Danderyd University Hospital, Stockholm, Sweden; j Department of Medical specialties, Danderyd University Hospital, Stockholm, Sweden

**Keywords:** Pulse wave velocity, HIV, Arterial stiffness, Sub-saharan africa

## Abstract

• Chronic HIV related inflammation and immune activation is independently associated with increased arterial stiffness.
• Majority of people living with HIV are found in Sub-Saharan Africa however, few studies have assessed arterial stiffness in this population.
• A prevalence (15.4%) of arterial stiffness was found in a population-based sample of 370 participants in Rakai, South Western Uganda.
• HIV infection significantly increased arterial stiffness.

## Graphical abstract

**Figure:**
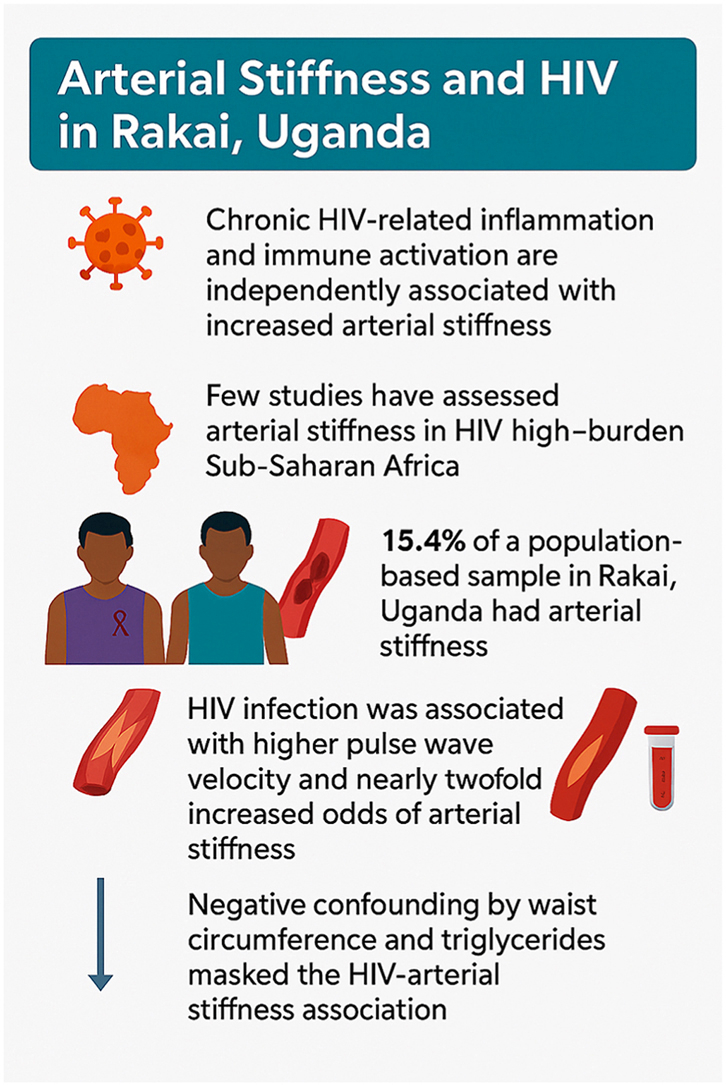

## 1 Introduction

Human Immunodeficiency Virus (HIV) has transitioned from a fatal infection to a chronic manageable condition, largely due to widespread access to antiretroviral therapy (ART). As life expectancy among people living with HIV (PLWH) continues to improve, long term complications including cardiovascular disease (CVD) have emerged as a growing concern [1]. CVDs are now a major contributor to mortality and morbidity among PLWH [[2], [3], [4]], with the global burden of HIV-related CVDs increasing nearly three-fold over the past two decades [5,6]. Studies, mostly from high-income countries, suggest that PLWH are approximately twice as likely to develop CVDs compared to HIV-negative individuals [6]. Proposed mechanisms include chronic immune activation, systemic inflammation, endothelial dysfunction and ART related metabolic changes [[7], [8], [9]].

Arterial stiffness, most commonly assessed by pulse wave velocity (PWV), is a non-invasive surrogate marker of vascular stiffening and subclinical organ damage [10]. PWV, which provides information on the structural characteristics of an individual's arteries has been validated as a reliable indicator of arterial stiffness and is increasingly used in clinical practice to assess cardiovascular risk [11,12]. This enables early detection of preclinical vascular disorders before they become clinically established. Elevated arterial stiffness has been consistently associated with increased risk of hypertension, stroke, heart failure and all-cause mortality, independent of traditional risk factors [13,14]. In addition, it predicts future cardiovascular events and mortality [12,15]. PWV has therefore become an important tool in cardiovascular risk stratification and could be used in clinical practice to detect preclinical vascular compromise.

Emerging evidence indicates that HIV infection may accelerate arterial stiffness through a complex interplay of viral effects, immune activation, and treatment-related factors [[16], [17], [18]]. Studies from high income countries report high prevalence of arterial stiffness among PLWH [7,11]. Systematic reviews and meta-analyses have demonstrated that PLWH exhibit higher arterial stiffness compared to HIV-negative individuals, even after adjustment for conventional risk factors [16,19]. However, the magnitude and clinical relevance of this association remain incompletely understood, particularly in Sub-Saharan Africa (SSA) where both HIV and CVD risk factors are highly prevalent [3,20].

Understanding the relationship between HIV status and PWV in SSA populations could support earlier detection of vascular compromise, improve cardiovascular risk stratification and inform tailored prevention strategies, to avert progression to overt CVD among PLWH [13,21]. In Uganda, few studies have evaluated arterial stiffness using PWV or accounted for local cardiovascular risks [22,23]. We thus sought to investigate the association between HIV infection and arterial stiffness using PWV in a population-based cohort in Rakai, South Western Uganda. Findings may help contextualize CVD risk in HIV care and support the integration of vascular screening in SSA settings.

## 2 Methods

### 2.1 Study design and setting

We conducted a matched cross-sectional study nested in the Rakai Community Cohort Survey (RCCS) between April 2024 and March 2025. The RCCS is an open, population-based cohort survey established in 1994 and comprised of ∼18,000 adults in 34 communities in Rakai, South Western Uganda [24]. The cohort is maintained through regular census and survey rounds conducted approximately every 18 to 24 months, during which trained field teams enumerate all eligible residents aged 15–49 years (recently expanded to include all those ≥15 years) and invite them to participate in structured interviews and biological sample collection. To date, 20 rounds of the survey have been conducted. The cohort is characterized by high participation, response and retention rates with robust community engagement, supported by longstanding partnerships with local health authorities and community advisory boards. For this study, we leveraged the RCCS infrastructure to identify and enroll HIV-positive and HIV-negative adults matched on age, sex, and community of residence, enabling a balanced comparison of arterial stiffness across HIV status within a well-characterized population.

### 2.2 Study population and eligibility criteria

**Fig. 1:**
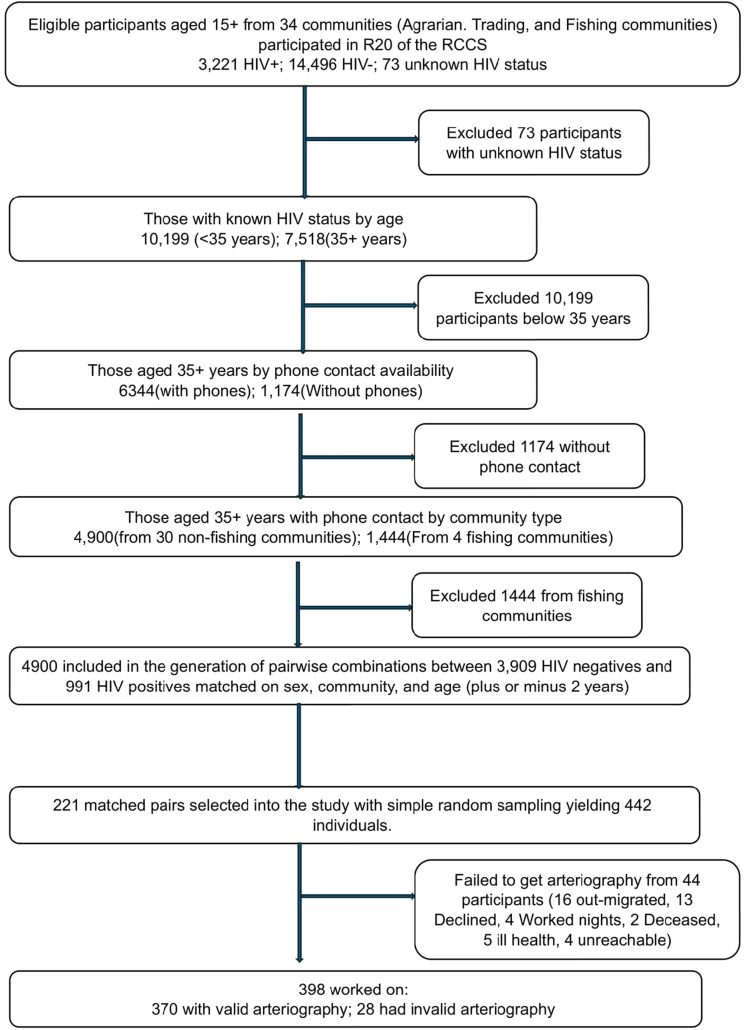
**Participant flow through the study.** Numbers represent individuals at each stage and reasons for exclusion are shown at each step.

Our target population were participants from the 20th survey round aged ≥35 years. Participants who were willing to comply with scheduled visits, study procedures and provided written informed consent were eligible for this study. We excluded participants who were mentally ill, critically ill, had no phone contact, no HIV test results, and/or were from fishing villages (hard to reach due to their high mobility) (see Fig. 1).

### 2.3 Sample size

Based on prior data from a pilot study in Rakai, South Western Uganda [22] and using the formula for sample size calculation for cross-sectional studies with continuous outcomes [25] and STATA 19 software we estimated that a sample size of at least 352 participants (176 per group) would provide 80% power to detect a 0.3 m/s difference in pulse wave velocity between the 2 groups with 5% alpha level and assuming a standard deviation of 1 m/s.

### 2.4 Sampling procedures and data collection

We used a random computer-generated code to select PLWH participants and their matched HIV negative counterparts using simple random sampling from a list of all participants in the 20th RCCS survey round. Using a phone number on record, a research assistant contacted and visited the home of each of the participants to conduct arterial stiffness measurement.

HIV status in the RCCS is determined through routine serological testing during each survey round. Blood samples are collected from participants and tested using Uganda Ministry of Health-approved guidelines. Results are linked to individuals via unique identifiers and securely stored in the RCCS database. For this study, HIV status was retrieved from the most recent RCCS round prior to enrollment. Newly diagnosed cases were included if confirmatory testing was completed. Duration of HIV infection and ART were retrieved from the survey data base.

Arterial stiffness was assessed using an arteriograph (Tensiomed™ Ltd, Budapest, Hungary) to measure PWV. Arm circumference was captured in the seated position before the start of arterial function measurements to determine the appropriate arm cuff for the measurement. The distance between the jugulum and symphysis was captured while the participant was in a standing position. Three measurements were taken after 10 min of rest and the average of results with a standard deviation < 1 (valid results) used for analysis. Arterial stiffness was assessed both as a continuous outcome and as a cut-off defined as a PWV ≥10.0 m/s, based upon current guidelines and recommendations [26,27]. Arterial function parameters from the arteriography included Aortic Augmentation Index, Return Time, and PWV.

Covariates and clinical measures: Other variables were extracted from the RCCS survey database. These included sociodemographic characteristics like age, sex and cardiovascular risk factors like history of ever using alcohol, or smoking cigarette, raw salt intake (yes if they add raw salt to meals and no if they do not), ART history (on treatment or not, ART duration) and HIV duration for PLHW, waist circumference, waist to hip ratio, height and weight to determine body mass index (BMI), lipid panel, mean 24-h systolic and diastolic blood pressure (extracted from ambulatory blood pressure measurements in which participants wore a monitor for over a 24-h period).

### 2.5 Statistical analysis

We examined the distributions of socio-demographic, behavioral and clinical characteristics by HIV status. Categorical variables were summarized using counts and percentages while numerical variables were summarized using means and standard deviations. Chi-square and student t-test were used to examine bivariate differences in categorical and continuous variables, respectively.

Next, we computed the proportion of participants with arterial stiffness among PLWH and those without and compared the differences using chi-square test.

To determine the association of HIV status with the average continuous PWV values, we fitted crude and multivariable linear regression models with HIV as the independent variable. Covariates adjusted for included social-demographic characteristics (e.g., age, sex) and clinical characteristics (e.g., waist to hip ratio, low density lipoproteins, high density lipoproteins type c, cholesterol and triglycerides selected based on biological and statistical relevance). In addition, we used logistic regression to assess association of HIV status (exposure) with binary arterial stiffness (outcome).

Interaction was assessed by forming two-way interaction terms between the main exposure variable (HIV status) and other covariates, and then performing the likelihood ratio test. No significant interactions were found. Confounding was assessed using a directed acyclic graph (DAG) and by comparing the adjusted (HIV status with an additional variable (potential confounder)) and crude measures of association for HIV status in the models. A variable was considered a confounder if it caused a change of ≥10% in the estimate effects of HIV status and or is a known confounder from literature. Statistical significance was set at a p-value <0.05.

Lastly, a subgroup analysis was conducted exclusively among PLWH to evaluate the association between years of HIV infection on PWV and arterial stiffness. We conducted a one-way analysis of variance (ANOVA) to compare mean PWV values across predefined HIV duration groups. Participants were grouped into three categories based on years living with HIV: <10 years, 10–20 years, and >20 years. For ART duration, participants with missing values were assigned the group mean and thereafter grouped in two groups <10 years and >10 years. Logistic regression was used to assess association of HIV duration (exposure) with binary arterial stiffness (outcome). Data was analyzed using STATA version 19.0 (Stata Corp, College Station, Texas, USA).

### 2.6 Ethical considerations

Ethical permission was sought from the Uganda Virus Research Institute Research and Ethics Committee (UVRI-REC GC/127/987) and the Uganda National Council for Science and Technology (UNSCT HS3209ES). Participants provided written informed consent before any study-specific activity was performed. This study was performed in line with the principles of the Declaration of Helsinki. The authors confirm that the ethical policies of the journal, as noted on the journal's author guidelines page, have been adhered to.

## 3 Results

### 3.1 Participant characteristics

We first evaluated baseline characteristics of participants by HIV status. A total of 370 participants were enrolled including 178 (48.1%) PLWH. The mean age of the participants was 45.4 (SD = 6.6), with 66.5% female representation. Compared to the HIV negative participants, PLWH had significantly lower BMI and triglycerides. (Table 1).

**Table 1:** Baseline characteristics of participants by HIV status in Rakai, South Western Uganda.

Variable	Total	*PLWH*	*HIV Negative*	*p-Value*
	N = 370	N = 178	N = 192	
Age, years (mean ± SD)	45.4 (6.6)	45.4 (6.6)	45.4 (6.7)	0.66
Female sex, n (%)	246 (66.5%)	118 (66.3%)	128 (66.7%)	0.94
Current smoker, n (%)	36 (9.7%)	17 (9.6%)	19 (9.9%)	0.91
Alcohol intake, yes, n (%)	188 (50.8)	82 (46.1%)	106 (55.2%)	0.08
Raw salt intake, yes, n (%)	240 (64.9%)	113 (63.5%)	127 (66.2%)	0.59
BMI, kg/m2 (mean ± SD) *	23.8 (4.3)	23.3 (4.0)	24.3 (4.5)	0.02 *
Waist circumference, cm (mean ± SD)	82.9 (10.3)	82.0 (9.3)	83.7 (11.1)	0.11
Waist to hip ratio, (mean ± SD)	0.9 (0.1)	0.9 (0.1)	0.9 (0.1)	0.42
Cholesterol, mmol/L (mean ± SD)	4.2 (1.1)	4.1 (1.1)	4.3 (1.2)	0.09
HDL, mmol/L (mean ± SD)	1.1 (0.4)	1.1 (0.4)	1.2 (0.4)	0.13
24-h systolic BP, mmHg (mean ± SD)	120.5 (14.6)	119.5 (15.5)	121 (13.6)	0.23
24-h diastolic BP, mmHg (mean ± SD)	76.8 (10.8)	76.8 (11.5)	76.9 (10.3)	0.93
LDL, mmol/L (mean ± SD)	2.4 (0.8)	2.3 (0.8)	2.4 (0.9)	0.15
Triglyceride, mmol/L (mean ± SD) *	1.4 (0.7)	1.3 (0.6)	1.4 (0.8)	0.03 *

PLWH: People Living with HIV, SD: Standard deviation BMI: Body Mass Index, cm: centimeters, ART: Anti-retroviral therapy, BP: Blood pressure, HDL: High density lipoprotein, LDL: Low density lipoprotein, eGFR: Estimated glomerular filtration rate, *Significant at p-value<0.05.

**Fig. 2:**
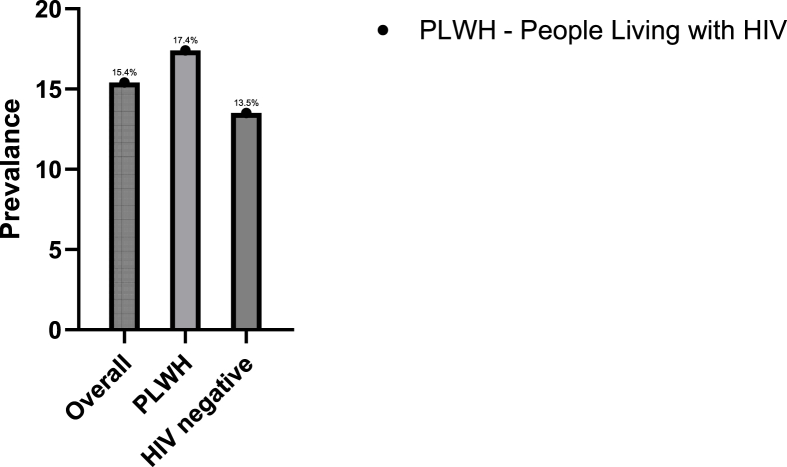
Prevalance of arterial stiffness among participants in Rakai, South Western Uganda.

Mean PWV was slightly higher among PLWH compared to HIV negative individuals but was not statistically significant ((8.6 ± 1.4 m/s Vs 8.4 ± 1.2 m/s, p-value = 0.2). Overall, the prevalence of arterial stiffness was 15.4% (95% CI = 12.1, 19.5). The proportion of participants with arterial stiffness was slightly higher among PLWH (17.4% Vs 13.5%) (Fig. 2 and Supplementary Table 1).

### 3.2 HIV association with PWV and arterial stiffness

We then assessed the association between HIV status, cardiovascular risk factors, and PWV as a continuous variable. In the adjusted linear regression, HIV infection was significantly associated with a 0.28 m/s higher PWV than those without HIV (adjusted β = 0.28, 95% CI: 0.02, 0.53). (Table 2). In addition, age, triglyceride levels, 24h Systolic BP and HDL-C were significantly associated with PWV in crude and adjusted models (Table 2)

**Table 2:** Linear regression models examining associations between HIV status, cardiovascular risk factors, and pulse wave velocity (PWV, continuous outcome) among participants in South Western Uganda.

PWV, m/s	Crude β Coefficient (95% CI)	p-Value	Adjusted β Coefficient (95% CI)	p-Value
PLWH	0.14 (−0.13, 0.41)	0.238	0.28 (0.003, 0.52) *	0.034
Age	0.03 (0.01, 0.05) *	0.001	0.02 (0.00, 0.04) *	0.039
Triglycerides	0.23 (0.04, 0.42) *	0.013	0.21 (0.01, 0.42) *	0.036
HDL, mmol/L	0.52 (0.18, 0.85) *	0.005	0.53 (0.15, 0.90) *	0.010
LDL, mmol/L	0.26 (0.11, 0.42)	0.002	0.14 (−0.03, 0.30)	0.121
Waist, cm	0.02 (0.00, 0.03) *	0.009	−0.00 (−0.01, 0.01)	0.873
Sex, female	0.08 (−0.20, 0.37)	0.582	0.10 (−0.18, 0.39)	0.426
24h Systolic BP	0.03 (0.02, 0.04) *	0.000	0.03 (0.02, 0.04) *	0.000

PLWH: People Living with HIV, PWV: Pulse wave velocity, HIV: Human Immunodeficiency Virus, HDL: High density lipoprotein, LDL: Low density lipoprotein. BP: Blood pressure *Asterisk denotes statistical significance (i.e., p < 0.05).

Next, we examined arterial stiffness defined categorically as PWV ≥10 m/s. HIV infection was associated with increased odds of arterial stiffness. While the crude association was not significant (OR = 1.34, 95% CI: 0.76, 2.37), the adjusted model showed nearly twofold higher odds among PLWH compared to HIV-negative individuals (AOR = 1.94, 95% CI: 1.01, 3.69, p = 0.045). (Table 3).

**Table 3:** Logistic regression models examining associations between HIV status and arterial stiffness, defined as PWV ≥10 m/s (categorical outcome) among participants in Rakai, South Western Uganda.

	Crude Odds Ratio (95% CI)	p-value	Adjusted Odds Ratio (95% CI)	p-value
PLWH	1.34 (0.76, 2.37)	0.303	1.94 (1.01, 3.69) *	0.045
Age, years	1.03 (0.99, 1.07)	0.156	1.01 (0.97, 1.04)	0.584
Triglycerides	1.19 (0.81, 1.76)	0.370	1.40 (0.82, 2.40)	0.215
HDL, mmol/L	2.36 (1.22, 4.54) *	0.010	4.04 (1.58, 10.31) *	0.003
LDL, mmol/L	1.71 (1.23, 2.38) *	0.002	2.24 (1.24, 4.03) *	0.007
Waist, cm	1.02 (0.99, 1.05)	0.185	1.00 (0.97, 1.04)	0.842
Sex, female	1.01 (0.55, 1.84)	0.975	1.06 (0.53, 2.13)	0.870
24h Systolic BP	1.04 (1.02, 1.06) *	0.000	1.03 (1.01, 3.70) *	0.001
Cholesterol, mmol/L	1.37 (1.06, 1.78) *	0.018	0.67 (0.43, 1.06)	0.087

PLWH: People Living with HIV, HDL: High density lipoprotein, LDL: Low density lipoprotein. BP: Blood pressure *Asterisk denotes statistical significance (i.e., p < 0.05).

### 3.3 Sub-group analysis among PLWH only

Finally, we explored subgroup analyses among PLWH. Arterial stiffness was more common among PLWH with HIV duration >20 years. The mean PWV was highest in the >20 years group (M = 9.13, SD = 1.58). ANOVA revealed a statistically significant difference in PWV across the HIV duration groups **(p = 0.009)**. (Supplementary Table 2).

HIV duration was not significantly associated with arterial stiffness after adjustment. Participants with 10–20 years of HIV showed a borderline protective effect in crude analysis (OR = 0.35, 95% CI: 0.12, 1.01, p = 0.053), but this was attenuated in the adjusted model (AOR = 0.58, 95% CI: 0.18, 1.92). Those with >20 years of HIV had higher odds of stiffness (AOR = 2.11, 95% CI: 0.69, 6.43), though not statistically significant. ART duration was not associated with stiffness. (Table 4).

**Table 4:** Univariate and multivariable logistic regression analysis of the association of HIV duration with arterial stiffness among participants in Rakai, South Western Uganda.

		Crude odds ratio (95% CI)	p-value	Adjusted Odds ratio (95% CI)	p-value
HIV duration	<10 years	Ref	Ref		
	10-20 years	0.35 (0.12, 1.01)	0.053	0.58 (0.18, 1.92)	0.373
	>20 years	1.97 (0.79, 1.02)	0.146	2.11 (0.69, 6.43)	0.188
ART years		0.96 (0.86, 1.08)	0.540	0.96 (0.82, 1.11)	0.583
Age, years		1.06 (1.00, 1.12)	0.042 *	1.03 (0.96, 1.11)	0.394
Sex, female		0.77 (0.34, 1.70)	0.518	0.98 (0.38, 2.52)	0.968
24h Systolic BP		1.03 (1.01, 1.06)	0.006 *	1.03 (1.00, 1.05)	0.049 *
HDL, mmol/L		2.16 (0.89, 5.25)	0.089	2.73 (0.83, 9.04)	0.100
LDL, mmol/L		1.59 (0.94, 2.70)	0.086	2.27 (0.82, 6.29)	0.115
Cholesterol, mmol/L		1.25 (0.86, 1.82)	0.240	0.64 (0.29, 1.38)	0.251
Triglycerides, mmol/L		1.40 (0.74, 2.65)	0.304	1.29 (0.56, 2.94)	0.552

BP: Blood pressure ART: Antiretroviral therapy, HIV: Human Immunodeficiency Virus. HDL: High density lipoprotein, LDL: Low density lipoprotein *Level of significance at p-value<0.005.

## 4 Discussion

In this population-based study from Uganda, arterial stiffness was observed in 15.4% of participants, representing a substantial burden in a relatively young cohort with a mean age of 45 years. The prevalence was slightly higher among PLWH, consistent with evidence that HIV infection contributes to vascular dysfunction beyond traditional risk factors. HIV infection was significantly associated with a 0.28 m/s higher PWV and nearly twofold higher odds for arterial stiffness compared to those without HIV.

We found that the highest proportion of participants with arterial stiffness were PLWH as has been reported in other studies [16,28]. These findings are also consistent with previous studies conducted in Uganda, that reported high prevalence of arterial stiffness, ranging from between 35% and 43%, although limited by smaller sample sizes [22,23]. In this study HIV infection was significantly associated with a 0.28 m/s higher PWV. In addition, HIV infection was associated with increased odds of arterial stiffness. Although the crude association did not reach statistical significance, adjustment for key covariates revealed nearly twofold higher odds of arterial stiffness among PLWH compared to HIV-negative individuals. Sequential modeling demonstrated that this shift from a non-significant crude association to a significant adjusted association was driven by negative confounding from waist circumference and triglyceride levels. PLWH in our cohort had lower waist circumference and triglyceride levels compared to HIV-negative individuals, which attenuated the crude HIV–PWV association. Once these protective factors were accounted for, the underlying relationship between HIV and arterial stiffness became evident. This highlights the importance of considering metabolic factors when evaluating vascular risk in PLWH, and illustrates how crude null findings may mask clinically relevant associations (Supplementary Table 3). This finding suggests that traditional risk factors may mask the contribution of HIV to vascular dysfunction, and that HIV-related mechanisms such as chronic immune activation, inflammation, and metabolic alterations likely play an independent role. This result aligns with prior studies reporting heightened vascular stiffness among PLWH [21]. Similarly in a recent (2021) systematic review and meta-analysis of 17 studies mostly from high income countries, arterial stiffness was found to be elevated overall in individuals with HIV relative to controls [16].

Notably, HDL cholesterol showed the strongest independent positive association with PWV and arterial stiffness, which is somewhat unexpected given its typically protective role in cardiovascular health. One possible explanation is that among PLWH, HDL particles may undergo structural and functional alterations due to chronic inflammation and ART exposure [[29], [30], [31]]. These changes could diminish HDL's protective effects or even contribute to vascular dysfunction [30]. Further investigation is warranted to explore whether qualitative differences in HDL composition or function among PLWH underlie this association. The association between HIV status and higher PWV/odds of arterial stiffness supports the hypothesis that HIV related processes including immune activation, chronic inflammation or ART-related metabolic effects induce structural remodelling of the vascular wall, characterized by, among other things: fibrotic deposition in the intimal and medial layers, medial smooth muscle cell apoptosis and necrosis, fragmentation and degradation of elastin fibers, calcific deposits within the extracellular matrix, and enhanced transvascular permeability facilitating macromolecular diffusion into the arterial wall, which collectively impair arterial elasticity and compliance, contributing to elevated PWV and arterial stiffness [[7], [8], [9]]. Conversely, waist circumference and sex were not significantly associated with PWV or arterial stiffness in this study, suggesting that body composition and sex-based physiological differences may play a less prominent role in vascular stiffness within this cohort, or that their effects are mediated by other included variables.

The duration of HIV infection may also influence the progression of arterial stiffness. In our study, PWV was significantly higher among individuals with more than 20 years of HIV infection (ANOVA p = 0.009), suggesting that prolonged HIV exposure may accelerate vascular stiffening. Although the association between HIV duration and arterial stiffness did not reach statistical significance in adjusted models, participants with over 20 years of HIV infection had more than twofold higher odds of arterial stiffness compared to those with less than 10 years. These findings highlight a potential cumulative effect of long-term HIV infection on vascular aging, warranting further investigation in larger cohorts. This aligns with prior research showing that chronic inflammation and endothelial dysfunction persist despite viral suppression, contributing to early vascular changes and increased cardiovascular risk [32,33]. In contrast, arterial stiffness was not associated with ART exposure in this study. This may reflect a stabilizing effect of long-term ART on vascular function or confounding by regimen type and adherence. Early initiation of ART as is the practice in Uganda since 2016 has been shown to be protective against progression of arterial stiffness [16] as it ameliorates the inflammatory processes. In our study, 94% of PLWH were on an ART treatment. In addition, well controlled HIV as evidenced by high ART adherence rates and viral load suppression in this cohort [24] could be responsible for the attenuation of the effects of the ART duration on arterial blood vessels. Future studies should model both HIV duration and duration of antiretroviral therapy (ART) against pulse wave velocity (PWV) to assess their independent and combined effects on arterial stiffness. This approach would help clarify the temporal relationship between HIV-related factors and vascular health, and may uncover whether longer exposure to ART contributes to stabilization or improvement in PWV among PLWH.

This study had several limitations. As a cross-sectional study, it cannot establish temporality or causality between risk factors and arterial stiffness. Furthermore, the study was conducted in a predominantly rural population in Rakai, Uganda, and the findings may not be generalizable to more urban towns and cities in the country. Nonetheless, strengths of the study include the population-based design and the matching of participants by age, sex and community which limited confounding. Additionally, exposure to traditional risk factors such as alcohol use and smoking was not significantly different between the groups. The strength and consistency of associations observed suggests a multifactorial pathophysiology of arterial stiffness in HIV. Longitudinal studies are needed to explore temporal relations and mechanistic pathways including the potential protective benefits of long-term ART.

## 5 Conclusion

In conclusion, this population-based study revealed that arterial stiffness is prevalent among adult participants in Rakai, South Western Uganda. Furthermore, PLWH exhibited significantly elevated PWV and odds for arterial stiffness, indicating increased vascular aging and cardiovascular risk.

## Authors’ contribution

CB developed the idea of the study, developed the analysis plan, conducted the analysis, and wrote the first draft of the manuscript, MSN and SN supported data collection, cleaning and analysis, ZZ, WC, GK, JK, PL, LC, HN supervised the research, data curation and analysis. All the authors have read and approved the final manuscript.

## Data statement

Data will be shared or made available by the corresponding author upon reasonable request.

## Declaration of generative AI and AI-assisted technologies in the manuscript preparation process

During the preparation of this manuscript, the author(s) utilized Microsoft Copilot to assist with grammar checking and to improve clarity and conciseness of the writing. Following the use of this tool, the author(s) carefully reviewed and edited all content, and accept full responsibility for the accuracy, integrity, and originality of the published article.

## Funding

Research reported in this publication was supported by the Fogarty International Center of the National Institutes of Health under Award Number D43TW011401. The content is solely the responsibility of the authors and does not necessarily represent the official views of the National Institutes of Health.

## Declaration of competing interest

The authors declare that they have no known competing financial interests or personal relationships that could have appeared to influence the work reported in this paper.
