# Supplementary material for: Association between HIV infection and arterial stiffness: A population-based cross-sectional study from Rakai, South Western Uganda
Source: Atheroscler Plus. 2026 Mar 20;65:100559. doi: 10.1016/j.athplu.2026.100559 (PMC13091412; doi:10.1016/j.athplu.2026.100559)
Supplement: Multimedia component 1 [file mmc1.docx]

**Supplementary Materials**

**Supplementary Table 1.** Arterial function measurements by HIV status among participants in Rakai, South Western Uganda

| Variable | Total N (%) | PLWH N (%) | HIV Negative N (%) |
| --- | --- | --- | --- |
| Pulse wave velocity, m/s (mean ± SD) | 8.5 (1.3) | 8.6 (1.4) | 8.4 (1.2) |
| Arterial stiffness, PWV ≥ 10m/s n (%) | 57 (15.4%) | 31 (17.4%) | 26 (13.5%) |
| Aortic augmentation index, % | 27.1 (13.2) | 26.9 (13.3) | 27.2 (13.2) |
| Return time, ms | 114.7 (16.3) | 114.5 (17.3) | 114.9 (15.4) |

*HIV: Human Immunodeficiency Virus, PLWH: People Living with HIV, SD: Standard deviation, PWV: Pulse wave velocity*

**Supplementary Table 2.** Arterial stiffness by HIV and ART duration among participants in Rakai, South Western Uganda

| Variable | HIV duration (years) | | | ART duration (years) | |
| --- | --- | --- | --- | --- | --- |
|  | <10 years | 10-20 years | >20 years | <10 years | >10 years |
| Pulse wave velocity, m/s (mean ± SD) * | 8.6 (1.4) | 8.3 (1.2) | 9.1 (1.6) | 8.8 (1.4) | 8.2 (1.1) |
| Arterial stiffness PWV ≥10m/s Yes, n (%)  No, n (%) | 11 (19.6%)  45 (80.4%) | 6 (7.9%)  70 (92.1%) | 14 (32.6%)  29 (67.4%) | 27 (20.0%)  108 (80%) | 4 (9.3%)  39 (90.7%) |

*PWV: Pulse wave velocity, SD: Standard deviation, ART: Antiretroviral therapy, HIV: Human Immunodeficiency Virus. *Significant at p-value<0.05*

**Supplementary Table 3.** Sequential modeling of HIV status and PWV (continuous outcome)

| Model | Covariates Included | β (95% CI) for HIV status | p-Value |
| --- | --- | --- | --- |
| 1 | Crude (no covariates) | 0.16 (-0.11, 0.42) | 0.238 |
| 2 | + 24h Systolic BP | 0.21 (-0.38, 0.47) | 0.096 |
| 3 | + 24h Systolic BP, HDL | 0.23 (-0.26, 0.48) | 0.079 |
| 4 | + 24h Systolic BP, HDL, LDL | 0.24 (-0.01, 0.50) | 0.056 |
| 5 | + 24h Systolic BP, HDL, LDL, Triglycerides | 0.28 (0.02, 0.53) | 0.032 * |
| 6 | + 24h Systolic BP, HDL, LDL, Triglycerides, Waist | 0.29 (0.03, 0.55) | 0.026 * |
| 7 | + 24h Systolic BP, HDL, LDL, Triglycerides, Waist, Age | 0.28 (0.21, 0.53) | 0.034 |
| 8 | + 24h Systolic BP, HDL, LDL, Triglycerides, Waist, Age, Sex | 0.28 (0.21, 0.53) | 0.034 |

*Pulse wave velocity, HIV: Human Immunodeficiency Virus, HDL: High density lipoprotein, LDL: Low density lipoprotein. BP: Blood pressure *Asterisk denotes statistical significance (i.e., p<0.05)*
